# All-fiber spatial rotation manipulation for radially asymmetric modes

**DOI:** 10.1038/s41598-017-02781-2

**Published:** 2017-05-31

**Authors:** Qi Mo, Zhikun Hong, Dawei Yu, Songnian Fu, Liang Wang, Kyunghwan Oh, Ming Tang, Deming Liu

**Affiliations:** 1 Wuhan National Laboratory for Optoelectronics, and School of optical and electronic information, Huazhong University of Science and Technology, Wuhan, 430074 China; 2 Wuhan Research Institute of Posts and Telecommunications, 88# Youkeyuan Road, Hongshan District, Wuhan 430074 China; 3 Department of Electronic Engineering, The Chinese University of Hong Kong, Hong Kong, Hong Kong; 4 Photonic Device Physics Laboratory, Department of Physics, Yonsei University, Seoul, 120-749 South Korea

## Abstract

We propose and experimentally demonstrate spatial rotation manipulation for radially asymmetric modes based on two kinds of polarization maintaining few-mode fibers (PM-FMFs). Theoretical finding shows that due to successful suppression of both polarization and spatial mode coupling, the spatial rotation of radially asymmetric modes has an excellent linear relationship with the twist angle of PM-FMF. Both elliptical core and panda type FMFs are fabricated, in order to realize manageable spatial rotation of LP_11_ mode within ±360° range. Finally, we characterize individual PM-FMF based spatial orientation rotator and present comprehensive performance comparison between two PM-FMFs in terms of insertion loss, temperature sensitivity, linear polarization maintenance, and mode scalability.

## Introduction

Recently, few-mode fiber (FMF) has shown many remarkable performances in various applications, which are not possible in standard single mode fibers (SSMFs). For fiber optic communication, several modes can be launched into FMFs, enabling mode division multiplexing (MDM) to further increase transmission capacity limit set by SSMF^1–3^. For fiber-based devices, FMF-based sensors have drawn substantial attentions due to their inherently distinctive optical characteristics of higher order modes^4, 5^, which cannot be provided by radially symmetric fundamental mode in SSMFs. Generally, the true modes propagating over the circular-core FMF are vector modes^1, 6^. However, LP mode basis is commonly used because LP modes are more readily excited and detected than the vector modes. For l ≥ 1, LP_lm_ modes are radially asymmetric modes^7^ as the electric field distribution along the angular direction is divided into several segments. In other words, spatial distribution of LP_lm_ (l ≥ 1) modes varies with the different angle. Spatial rotation manipulation means that the mode intensity pattern keeps its original profile while the symmetry axis rotates. Due to the radially asymmetric distribution of LP_lm_ (l ≥ 1) modes, the need for spatial rotation manipulation is frequently encountered in both scientific research and engineering applications. For the phase plate or liquid crystal on silicon (LCOS) based mode division de-multiplexers^8, 9^, optimization of spatial orientation of radially asymmetric mode is necessary in order to realize mode selective conversion and reduce the computation complexity of multi-input multi output (MIMO) processing simultaneously. Additionally, spatial rotation capability for radially asymmetric modes can provide a versatile tool for optical trapping and tweezers applications^10, 11^. Recently, generation of cylindrical vector beam (CVB) by coherent superposition of two spatially orthogonal LP modes with high polarization purity has been demonstrated^12^. Fiber specklegram sensors (FSS) using LP_21_ mode rotation^13^ and orbital angular momentum (OAM) generation by two spatially orthogonal LP modes with fixed phase delay^14, 15^ have been also reported. For those applications, fine and reliable adjustment of the spatial orientation of high order modes has become a basic necessity. Until now, only the LP_11_ mode rotator based on the planar lightwave circuit (PLC) technique has been demonstrated with an insertion loss (IL) of less than 0.46 dB over the spectral range from 1450 nm to 1650 nm^16^. The PLC-based LP_11_ mode rotator is fabricated with a trench structure. The spatially symmetrical axes of LP_11_ mode can be rotated with 45 or 135 degrees by properly setting those trench parameters. If LP_11a_ (LP_11b_) mode with an 90 degrees (or 0 degree) symmetrical axes is coupled to such PLC-based rotator, two orthogonal LP_11_ modes whose symmetrical axes are 45 and 135 degrees are equally excited and propagated with different propagation constants *β*_1_ and *β*_2_. By choosing the waveguide length as half beat-length, which is defined as π/(*β*_1_ − *β*_2_), the initially launched LP_11a_ (LP_11b_) mode can be rotated into LP_11b_ (LP_11a_) mode. We can see that the PLC-based mode rotator must agree well with a half beat length, for the purpose of successful mode rotation. In particular, the PLC-based mode rotator can only be applied to LP_11_ mode, because the beat length value is different for individual high order modes. Furthermore, the PLC-based mode rotator can only achieve a fixed rotation between LP_11a_ and LP_11b_ mode. The coupling efficiency between the on-chip device and optical fiber is still challenging, due to the mode field mismatch between the on-chip device and optical fiber. Thus, all-fiber mode orientation rotator has been investigated, but the implementation to realize a fixed angle rotation has been technically challenging^17^. Recently, 180° spatial orientation rotation of the LP_11_ mode has been reported by introducing a twist-induced mode coupling to traditional two-mode fiber^18^. Since guided modes in commercial FMF (c-FMF) are the vector modes with different propagation constants, spatial pattern of LP_lm_ (l > 0) mode varies periodically as they propagate and therefore they are also called pseudo-modes^19^. Thus it is very challenging to control the mode rotation manipulation in c-FMF. And all the proposed rotation schemes^16–18^ are either restricted to specific design parameters and fixed rotation angle or non-universal for all higher order radially asymmetric modes.

In this submission, we accomplish arbitrary spatial rotation manipulation of LP_11_ mode by two types of polarization maintaining few-mode fibers (PM-FMFs). Our theoretical finding shows that all-fiber arbitrary spatial rotation can be achieved by suppression of both spatial and polarization mode coupling. Thus, both elliptical core FMF (e-FMF) and panda type FMF (panda-FMF) with capability of both spatial mode and polarization maintenance are fabricated. Arbitrary rotation manipulation of LP_11_ mode within ±360° range is experimentally demonstrated by both two PM-FMFs with an easy implementation. Finally, we characterize the PM-FMF based spatial orientation rotator and for the first time, a thorough performance comparison between two PM-FMFs in many aspects, including the IL over different operation wavelength, temperature sensitivity, linear polarization maintenance and spatial pattern compatibility with common circular core FMF is presented.

## Results

**Figure 1:**
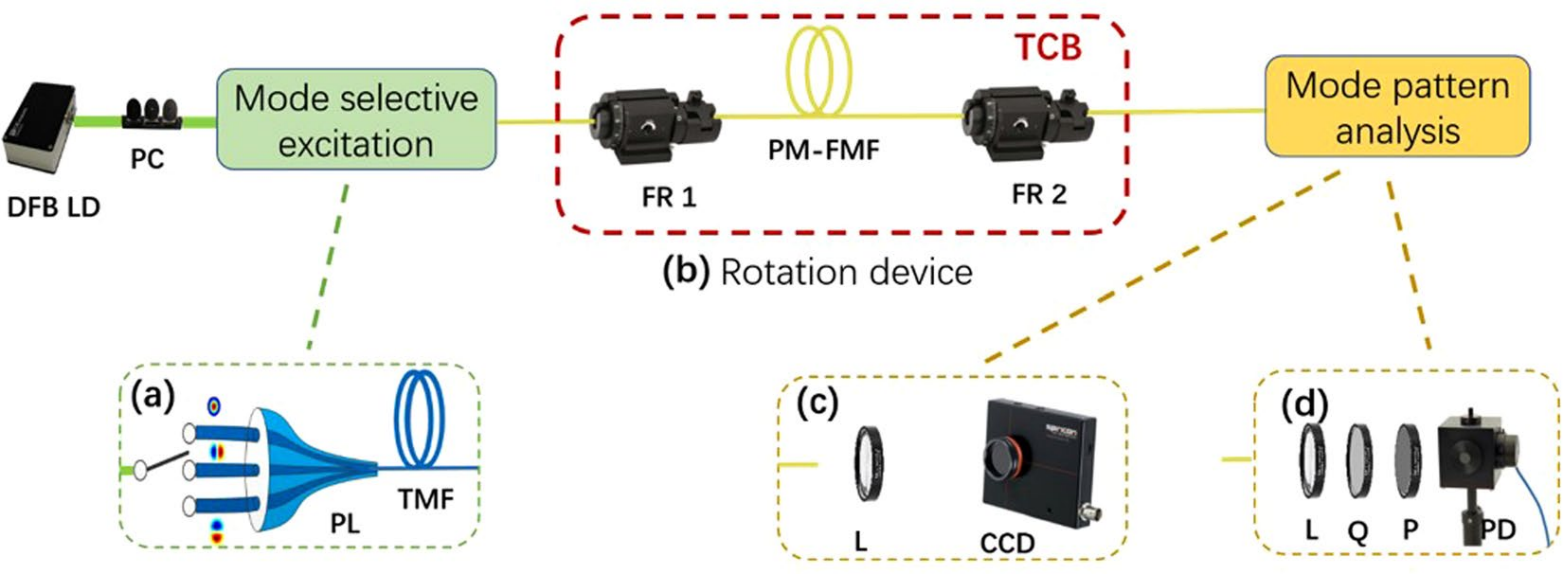
Experimental setup. (**a**) selective excitation of LP_11_ mode based on PL; (**b**) rotation device composed by two FRs and one section of PM-FMF; (**c**) mode pattern captured by CCD, and (**d**) power measurement. FR: fiber rotator; PL: photonic lantern; L: lens; Q: quarter-waveplate; P: polarizer; TCB: temperature control box.

**Figure 2:**
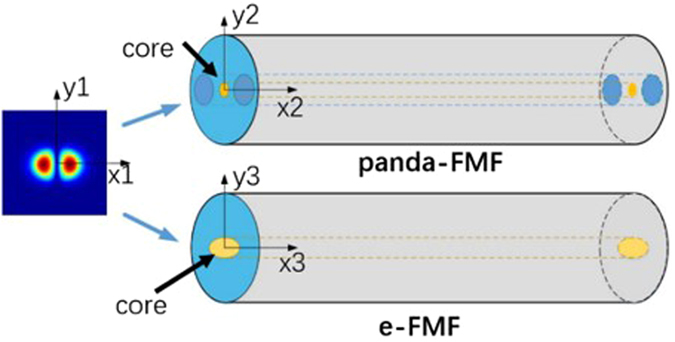
Axis of radially asymmetric modes agree with the symmetrical axis of the PM-FMFs

We experimentally examine the characteristics of arbitrary mode rotation by two PM-FMFs. The experimental setup is presented in Fig. 1. The output of a distributed feedback (DFB) laser diode (LD) (Yenista Optics OSICS-DFB), whose operation wavelength is 1550 nm, is coupled to one input port of the mode selective PL (Phoenix Photonics 3PL-0160153). The PL is a three-input mode excitation device which can be used to selectively convert the input fundamental modes to LP_01_, LP_11a_, and LP_11b_ modes, respectively. After the orientation optimization, the PM-FMF is fusion spliced to the output end of the PL by the polarization maintaining fiber fusion splicer (Fujikura FSM 100 P+). As a result, LP_11a_ mode is selectively excited. The two ends of the PM-FMF are respectively fixed to fiber rotator (FR, Thorlabs HFR007) whose angle increment is 5°. The total length of PM-FMF between two FRs is only 12 cm. By manually adjusting the second FR, we are able to manage the fiber twist angle with 2° precision. Mode pattern at the PM-FMF output is captured, in order to carry out both spatial distribution characterization and power measurement. Specifically, spatial distribution is recorded by a collimation lens and an infrared CCD (OPHIR Photonics SP620U-1550). The power measurement is accomplished by a collimation lens and integrating sphere (New Port Power Meter 1918-R with detector 819D-IG-2-CAL). The rotation device including two FRs and a section of PM-FMF is put into a self-fabricated temperature controlled box for temperature-dependent performance investigation. In order to realize pure mode rotation, only single mode is allowed to be launched into PM-FMFs each time, because the power spatial distribution of the mode basis possesses certain orientation with respect to the symmetrical axis of PM-FMF, as shown in Fig. 2. The propagation constants of those modes differ from each other. Otherwise, there will be an evolution of output mode pattern^20^.

### Arbitrary rotation of LP_11_ mode

**Figure 3:**
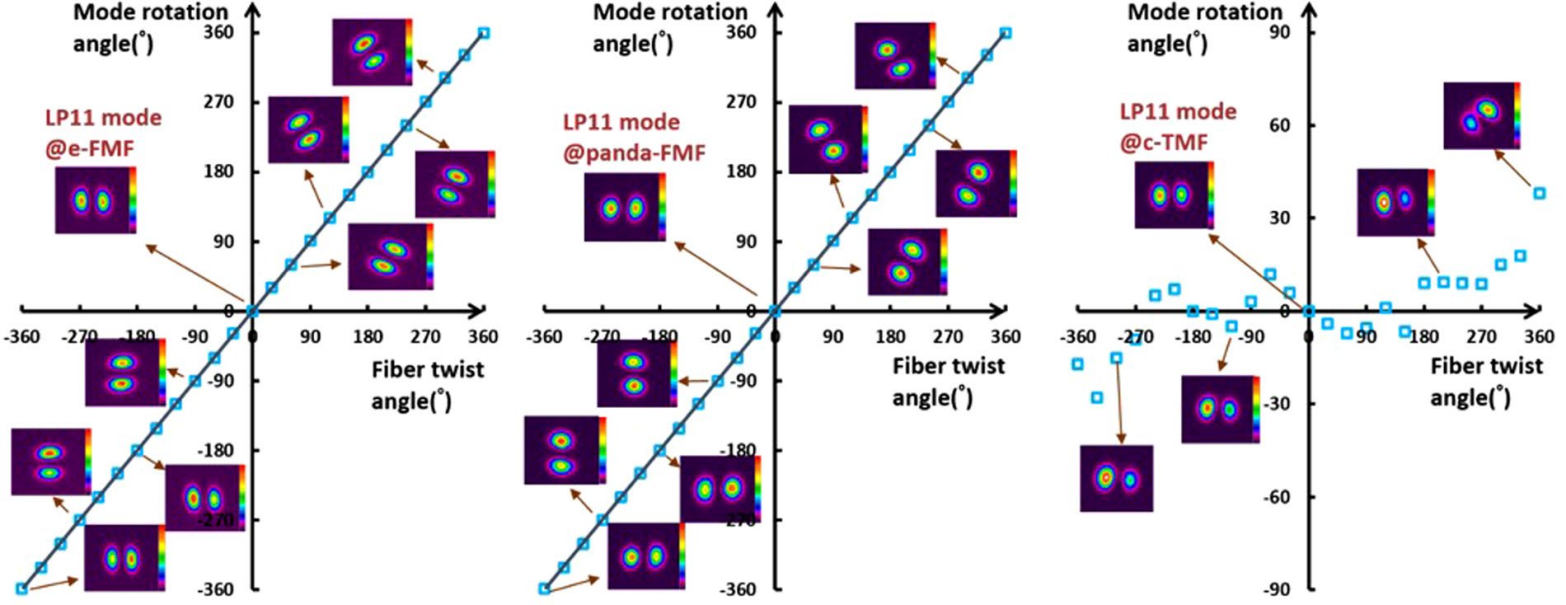
Spatial mode rotations from −360° to 360° of LP_11_ mode using e-FMF, panda-FMF, and commercial TMF, respectively.

First we launch LP_11_ mode into two PM-FMFs, respectively, and implement the fiber twisting with the help of the FR2. To make sure only one mode is launched, polarization maintaining fiber fusion splicer is used to rotate the PM-FMF input end to optimize orientation before splicing. When only one mode is launched and the PM-FMF is manually perturbed, no distortion of the captured mode pattern is observed. Next, the rotation angle of FR 2 is recorded, together with the captured output mode field. For the ease of comparison, LP_11_ mode rotation by twisting the commercial two-mode fiber (TMF, OFS) is also recorded. The results are summarized in Fig. 3.

### Performance comparison between two PM-FMFs

In order to provide a quantitative comparison of spatial mode rotation between the commercial TMF (c-TMF) and two PM-FMFs, rotation efficiency^21^ between the captured mode profile at PM-FMF output and the corresponding ideal LP mode profile with designated orientation angle are given by

1 $${\eta }_{modej}=\frac{{|{\iint }_{S}{E}_{capture}(x,y)\cdot {E}_{pure\_modej}^{\ast }(x,y)dxdy|}^{2}}{{{\iint }_{S}|{E}_{capture}(x,y)|}^{2}dxdy{{\iint }_{S}|{E}_{pure\_modej}(x,y)|}^{2}dxdy}$$

**Figure 4:**
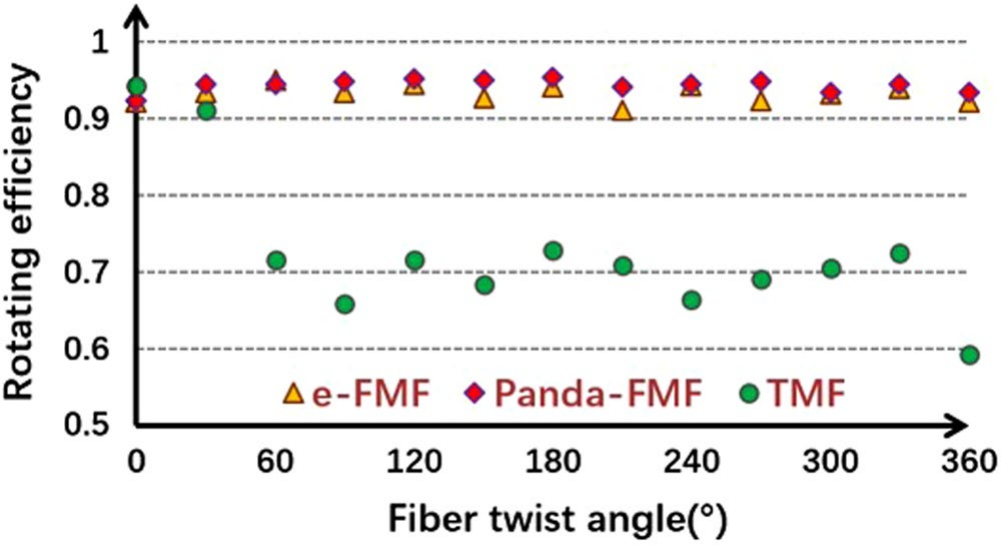
Rotating efficiency comparison of LP_11_ modes arising using e-FMF, panda-FMF, and commercial TMF.

**Figure 5:**
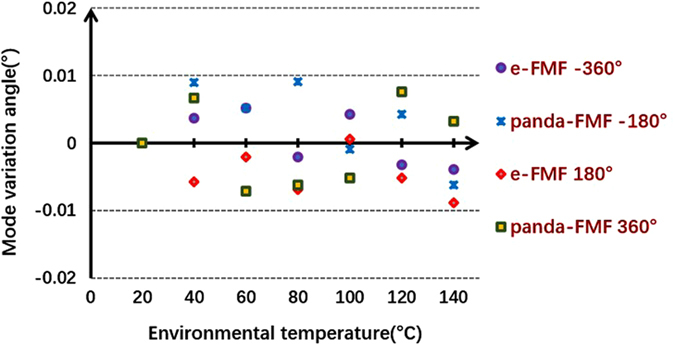
Mode rotation variation with respect to the temperature.

**Figure 6:**
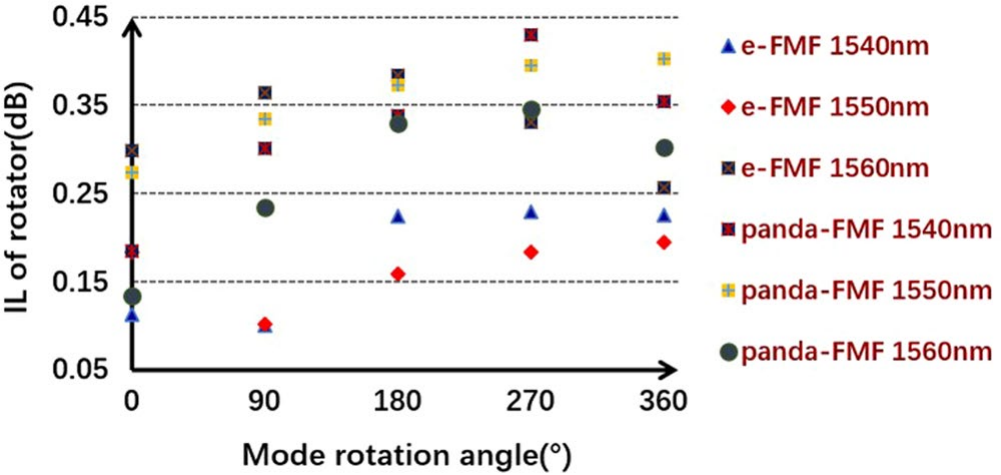
IL of all-fiber mode rotator with respect to the spatial rotation angle.

**Figure 7:**
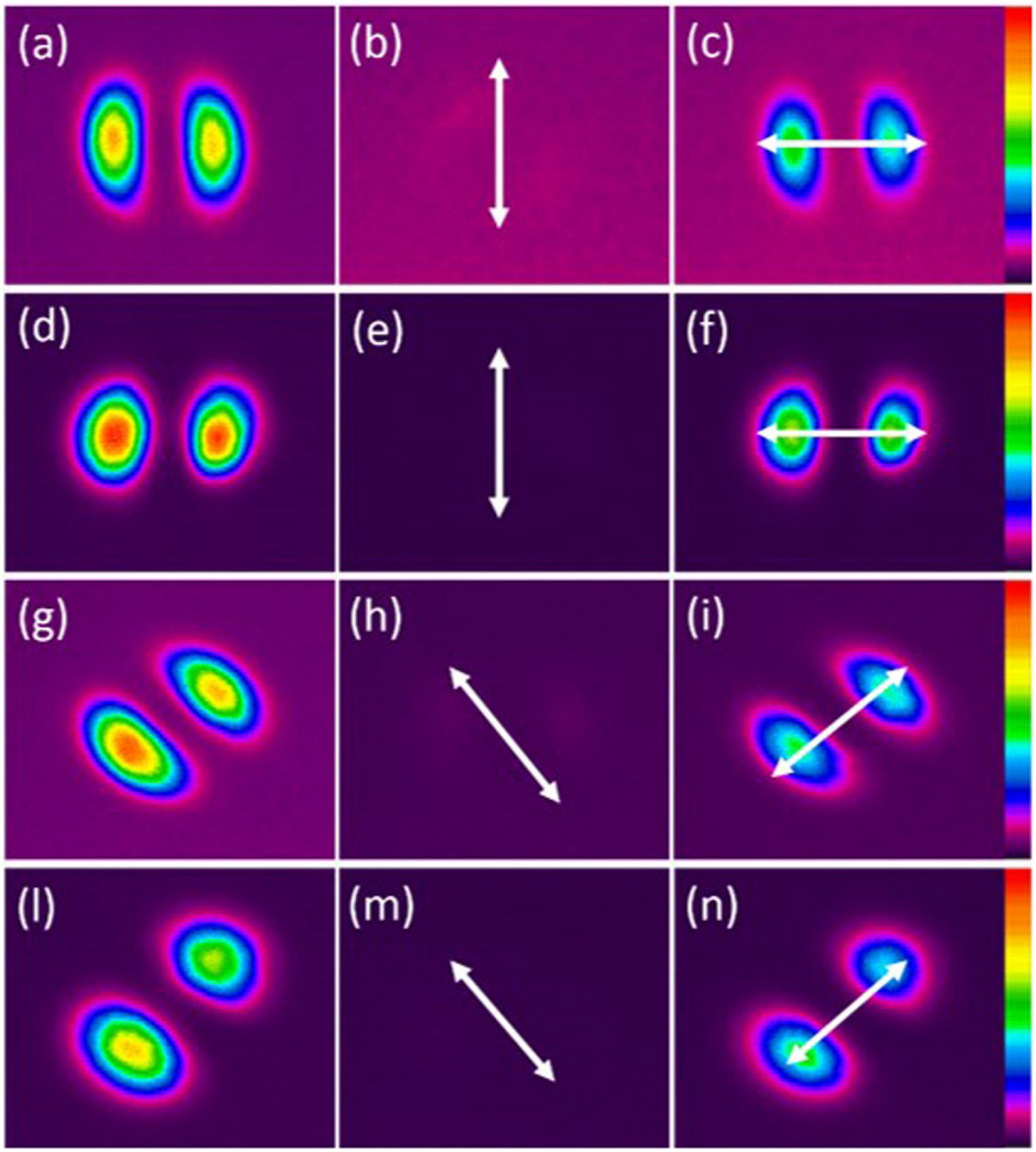
Captured LP_11_ mode from (**a**–**c**)/(**g**–**i**) e-FMF, and (**d**–**f**)/(**l**–**n**) panda-FMF with polarizer at two orthogonal directions. The arrows indicate the direction of the polarizer’s axis.

**Figure 8:**
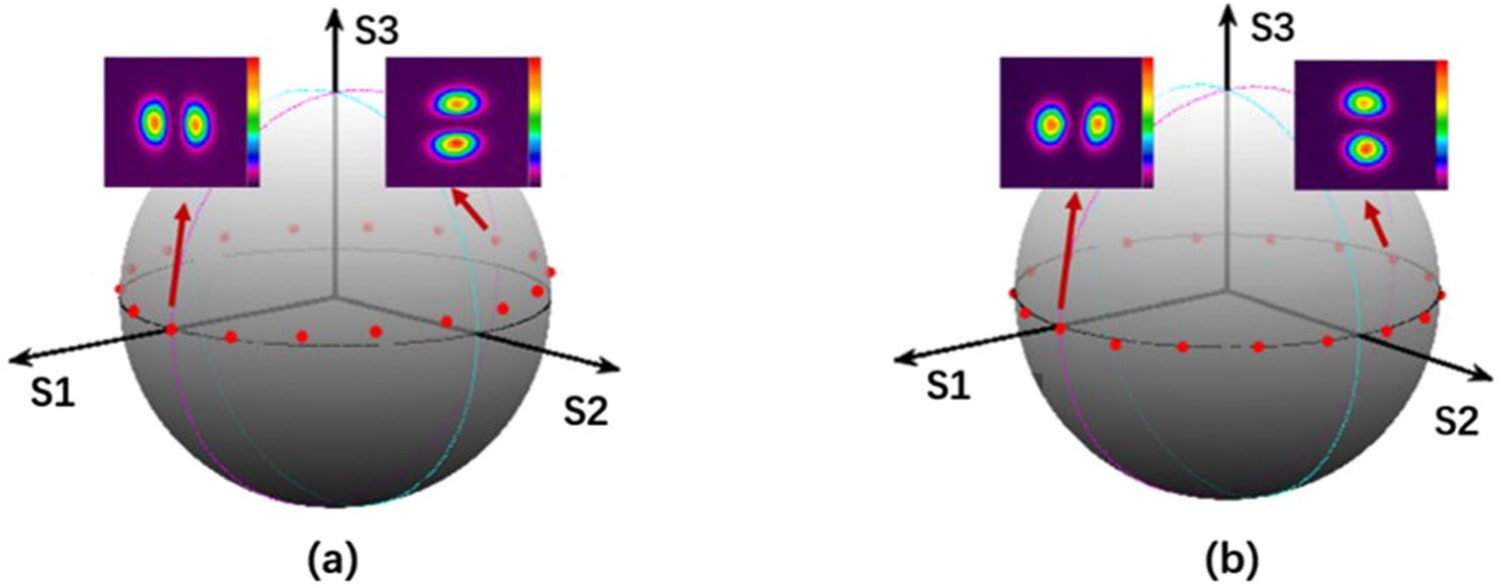
SOP of LP_11_ mode from (**a**) e-FMF, and (**b**) panda-FMF with different rotation angles.

The output electric field of PM-FMF E_*capture*_ is obtained by the combination of the captured mode intensity distribution with fixed phase difference between adjacent lobes. The ideal LP mode distribution E_*pure*_ can be achieved according to the diffraction theory^22^. Then, the overlap integral between E_*capture*_ and E_*pure*_ can be a good indicator of rotating efficiency after spatial rotation manipulation. As shown in Fig. 4, the mode rotating efficiency of LP_11_ mode by using two types of PM-FMFs is above 0.9. We believe that the little degradation is mainly due to the imperfect mode excitation at the input of PM-FMF. However, the mode rotating efficiency by using the commercial TMF degrades to 0.7. Because of severe mode coupling between degenerate modes arising in commercial TMF, the spatial rotation manipulation by commercial TMF is impossible. In order to investigate the temperature-dependent performance of all-fiber spatial mode manipulation, spatial rotation of LP_11_ modes using two PM-FMFs at four specific twist angles (−360°, −180°, 180°, and 360°) are individually measured, when the temperature is varied from 20 °C to 140 °C. In each twist angle measurement, only the environmental temperature is varied and all the other components are kept stable. The variations of rotation angle as a comparison with initial results at 20 °C are recorded, as shown in Fig. 5. All fluctuations of rotation angles are below ±0.01°, indicating of temperature-insensitive operation of the proposed all-fiber spatial orientation rotator. Meanwhile, we characterize the IL of the proposed spatial mode rotator. We monitor the maximum variation of optical power with respect to the operation wavelength, when the LP_11_ mode are rotated within 360° range. The IL in comparison with the situation without mode rotation operation is summarized in Fig. 6. The IL is less than 0.45 dB for all modes within 360° range, when the operation wavelength is varied from 1540 nm to 1560 nm. Finally, the state of polarization (SOP) at the PM-FMF output is determined, because both elliptical core and panda-type fiber are commonly used for the purpose of polarization maintaining in the SMF design^23^. Thus, we carry out accurate measurement of the Stokes vector during the spatial rotation of LP_11_ mode with interval of 10°, based on a manually rotatable quarter-wave plate and a fixed linear polarizer^24^, as shown in Fig. 1(d). When the e-FMF is rotated, the polarization extinction ratio are 15.1 dB for LP_01_ mode and 14.0 dB for LP_11_ mode. When panda-FMF is used for spatial mode rotation, the corresponding polarization extinction ratio are 26.8 dB for LP_01_ mode and 21.0 dB for LP_11_ mode. The CCD-captured LP_11_ mode pattern, when the polarizer located at the PM-FMF output with two orthogonal linear polarizations, is shown in Fig. 7. For the ease of understanding the SOP characterization results, we also plot the results on Poincare sphere, as shown in Fig. 8. We can conclude that the rotated LP_11_ mode is still linearly polarized, and its linear SOP can also rotate the same value as that of spatial pattern.

## Discussion

As shown in Fig. 3, for both e-FMF and panda-FMF, the rotation of LP_11_ mode has an excellent linear relationship with respect to the twist angle of PM-FMF, indicating a flexible manipulation of arbitrary mode profile rotation. Performance comparison of the two PM-FMF has also been carried out in several aspects. By calculating overlap integral between the captured mode profile at PM-FMF output and the corresponding ideal LP_11_ mode profile with designated orientation angle, rotation efficiency of LP_11_ by two PM-FMF are obtained. The LP_11_ mode rotation efficiency for two PM-FMF are almost the same, showing a good advantage over commercial TMF. When environmental temperature is varied, the variations of rotation angle of LP_11_ mode by two PM-FMFs is negligible, indicating of temperature-insensitive operation for two types of PM-FMFs. The IL over an operation wavelength from 1540 nm to 1560 nm are also characterized. For both PM-FMF based mode rotators, the IL are less than 0.45 dB. However, the capability of linear polarization maintenance of panda-FMF is better than that of e-FMF. The linear polarization maintenance of e-FMF can be further improved by increasing the ellipticity^25^. However, considering the issue of fiber coupling and mode scalability, the mode profile of panda-FMF is more compatible with that of c-FMF because of its circular core. In a summary, all-fiber spatial rotation performance between e-FMF and panda-FMF are almost the same except the polarization maintenance capability. Thus, we do recommend the use of panda-FMF for the purpose of arbitrary mode rotation manipulation. In particular, the rotation of other radially asymmetric modes is expected, when the designated mode is solely guided over the proposed specialty FMFs. Normally, the increment of core size is helpful to guide more high order radially asymmetric modes.

## Conclusions

In conclusion, we are able to arbitrarily rotate LP_11_ mode orientation within ±360° range by two types of PM-FMFs. Theoretical finding shows that spatial rotation manipulation of high-order radially asymmetric modes is possible due to the suppression of both spatial and polarization mode coupling, when the symmetric axis of input radially asymmetric mode is aligned to the symmetric axis of PM-FMF. Finally, we fabricate both e-FMF and panda-FMF and carry out a thorough performance comparison between two PM-FMFs in many aspects, including insertion loss over different operation wavelength, temperature sensitivity and linear polarization maintenance capability. Finally, we recommend the use of panda-FMF for the purpose of all-fiber spatial rotation manipulation for arbitrary radially asymmetric modes.

## Methods

**Figure 9:**
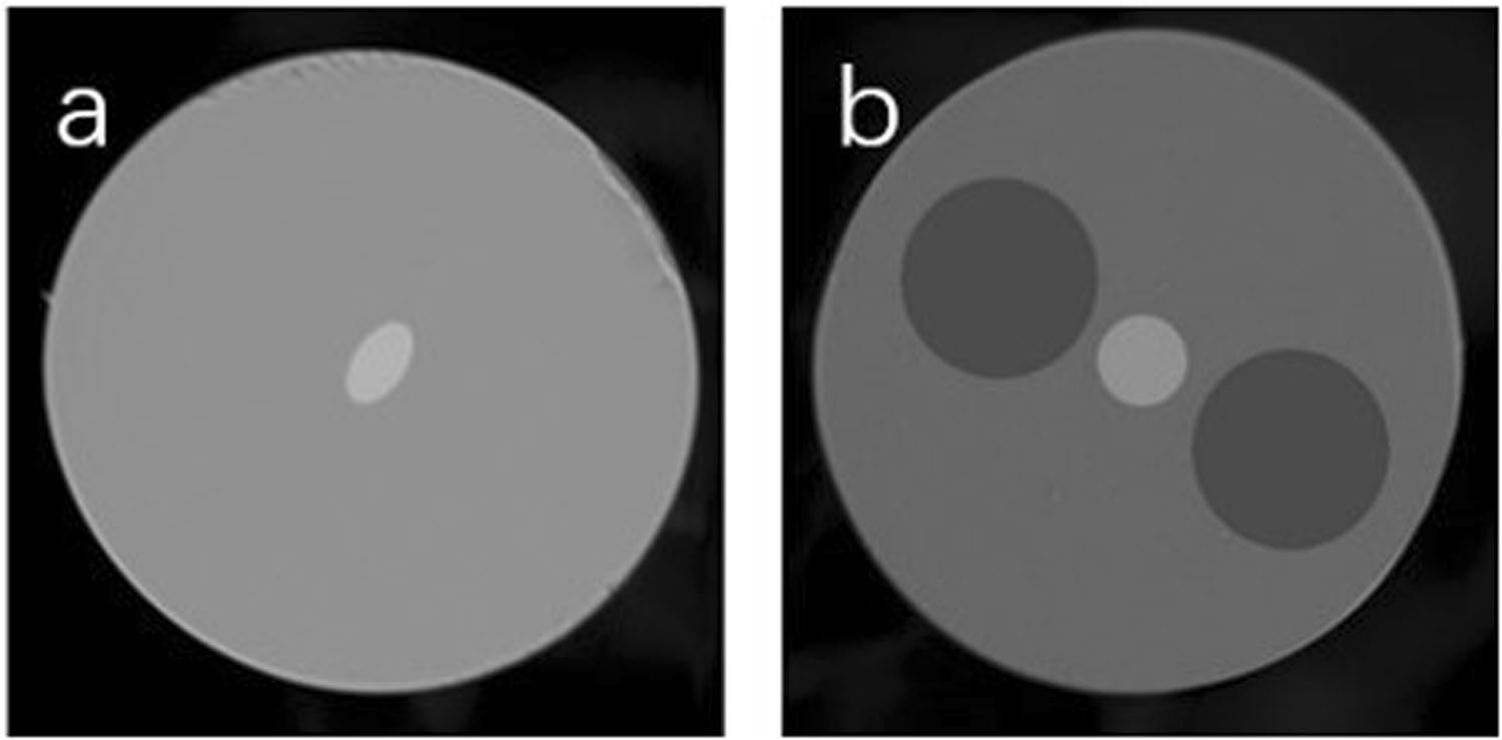
Captured SEM figure of (**a**) e-FMF, and (**b**) panda-FMF.

**Figure 10:**
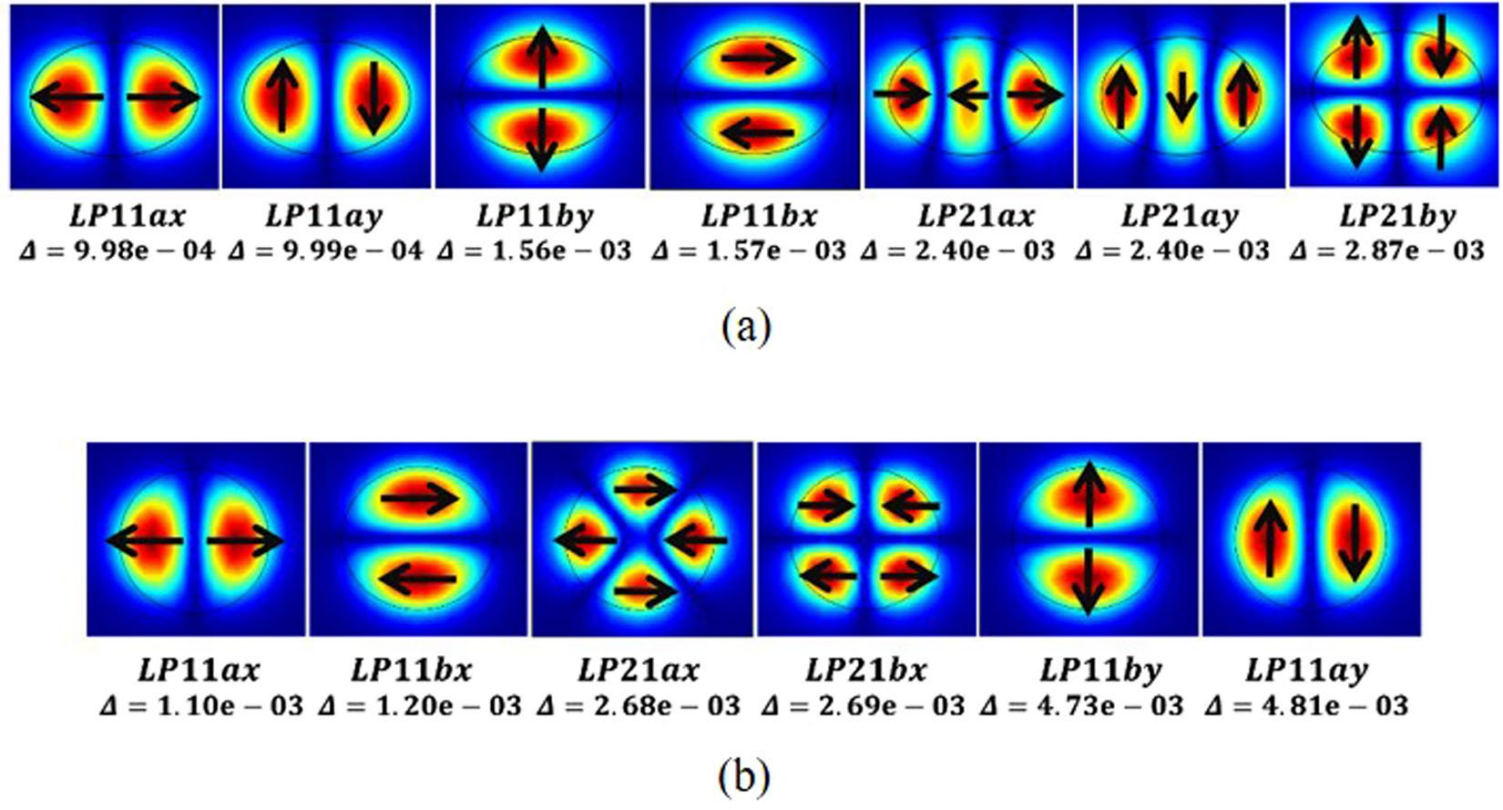
LP_11_ and LP_21_ mode profiles with mode spacing as comparison to LP_01_ mode arising in (**a**) e-FMF, (**b**) panda-FMF, respectively. The subscripts “a” and “b” represent individual spatial distributions, while subscripts “x” and “y” represent individual polarization directions.

Generally, mode propagation in the FMF can be described by coupled mode theory (CMT). Coupled mode equation can be solved analytically for a length of *z* in Jones matrix form^26^:

2 $$(\begin{array}{c}{{\boldsymbol{E}}}_{p}(z)\\ {{\boldsymbol{E}}}_{q}(z)\end{array})={e}^{-i{\beta }_{{\rm{0}}}z}[\begin{array}{cc}\cos (Sz)-i\frac{\delta }{S}\,\sin (Sz) & -i\frac{{k}_{pq}}{S}\,\sin (Sz)\\ -i\frac{{k}_{pq}}{S}\,\sin (Sz) & \cos (Sz)+i\frac{\delta }{S}\,\sin (Sz)\end{array}](\begin{array}{c}{{\boldsymbol{E}}}_{p}(0)\\ {{\boldsymbol{E}}}_{q}(0)\end{array})$$

where *E*_*p*_ (*0*) and *E*_*q*_ (*0*) are the electrical fields of two input adjacent vector modes. Mode coupling may occur between arbitrary two vector modes, but coupling between adjacent modes is usually the strongest. *β*_*0*_ = (*β*_*p*_ + *k*_*pp*_ + *β*_*q*_ + *k*_*qq*_)/*2* is the common propagation constant, *δ* = (*β*_*p*_ + *k*_*pp*_ − *β*_*q*_ − *k*_*qq*_)/*2* is the detuning factor while *β* = *2 π*/*λ* is propagation constants in vacuum, *β*_*p*_ = *2 πn*/*λ* is propagation constant of mode *p* arising in the FMF, n is the mode effective refractive index, and λ is operation wavelength. $S=\sqrt{({\delta }^{2}+{k}_{pq}^{2})}$ is the coupling strength, *k*_*pq*_ is inter-coupling coefficient and *k*_*pp*_ is the self-coupling coefficient under the designated LP mode group. Normally, the transmission matrix in Eq. (2) must be in close proximity to a unit matrix, in order to preserve the same output mode profile as the input one. Therefore, the off-diagonal elements must be small enough, regardless of the environmental perturbation, especially when the fiber is twisted. As a result, the coupling coefficient *k*_*pq*_ and coupling strength *S* need to be well managed by specifically enlarging the detuning factor *δ* and reducing the coupling coefficient *k*_*pq*_. Under the condition of fiber twisting, *k*_*pq*_ is determined by the following procedures. Generally, the transverse electric field of vector modes in the FMF can be described as:

3 $${{\boldsymbol{E}}}^{{\boldsymbol{t}}}(x,y,z)=\sum _{p}{A}_{p}(z){{\boldsymbol{E}}}_{p}^{{\boldsymbol{t}}}(x,y)$$

where *A*_*p*_ (*z*) = *a*_*p*_ *exp*(*iβ*_*p*_ *z*) is the mode amplitude of the FMF and *a*_*p*_ is the amplitude coefficient of mode *p* with propagation constant *β*_*p*_. When the FMF is twisted, the coupling coefficients becomes^27^

4 $${k}_{pq}=\frac{\iint [{\beta }^{2}{[{(\tilde{\varepsilon }{{\boldsymbol{E}}}_{q})}^{t}\times {{\boldsymbol{H}}}_{p}^{t\ast }]}^{z}+\beta {\beta }_{q}{{\boldsymbol{E}}}_{p}^{z\ast }{(\tilde{\varepsilon }{{\boldsymbol{E}}}_{q})}^{z}-i\beta {{\boldsymbol{E}}}_{p}^{z\ast }\nabla (\tilde{\varepsilon }{{\boldsymbol{E}}}_{q})]dxdy}{2{\beta }_{p}{\iint [{{\boldsymbol{E}}}_{p}^{t}\times {{\boldsymbol{H}}}_{p}^{t\ast }]}^{z}dxdy}$$

where ***E*** and ***H*** are corresponding electric and magnetic fields, superscripts *z* and *t* indicate the longitudinal and transverse field components, respectively. The asterisk * indicates complex conjugation. Then, the coupling coefficient *k*_*pq*_ between two vector modes can be determined. Here we take the coupling between TM_01_ and HE_21e_ modes in LP_11_ mode group as an example to explain rotation manipulation, when the fiber is twisted. By taking the mode coupling into account, the output mode field can be worked out according to Eq. (2) under rotation coordinate system. Meanwhile, the relationship between the stationary coordinate and the rotation coordinate system under the condition of fiber twisting can be described as^28^:

5 $$(\begin{array}{c}{{\boldsymbol{E}}}_{x}\\ {{\boldsymbol{E}}}_{y}\end{array})=(\begin{array}{cc}\cos ({\varphi }_{t}z) & -\,\sin ({\varphi }_{t}z)\\ \sin ({\varphi }_{t}z) & \cos ({\varphi }_{t}z)\end{array})(\begin{array}{c}{{\boldsymbol{E}}}_{\eta }\\ {{\boldsymbol{E}}}_{\xi }\end{array})$$

where *фt* is twisting rate. Hence, the electric fields at the stationary coordinate can be obtained, when the FMF is twisted. It is obvious that if the off-diagonal coefficients are small and Eq. (2) can be approximated as a unit matrix, there exists a prospect of manageable mode rotation of LP mode, when the FMF is twisted. In order to minimize off-diagonal coefficients, small coupling coefficient *k*_*pq*_ and a large detuning factor *δ* between propagated modes are required. In other words, mode spacing between propagated modes should be enlarged. However, for c-FMF, the coupling between adjacent vector modes within the designated LP mode group is severe^29^, so that corresponding transmission matrix in Eq. (2) cannot be approximated as a unit matrix. Consequently, the output mode profile after propagation over a section of c-FMF is random^6, 18^. Since e-FMF has been proposed for MIMO-less transmission with the mode spacing substantially enlarged^30, 31^, it might be a promising candidate for realizing mode rotation manipulation. We start to design and fabricate e-FMF first. The circular fiber preform is successfully fabricated by a method of plasma chemical vapor deposition (PCVD). Then, we carry out rod in tube (RIT) process after symmetrically grinding in order to obtain an elliptical shape. After that, the preform is drawn at the drawing speed of 200 m/min with a tension of 140 g. Finally, the e-FMF is ready for further characterization. The major and minor axes of elliptical core are 23 μm and 16 μm, respectively. The diameter of cladding is 121 μm while the refractive index (RI) difference between core and cladding is 3.9 × 10^−3^. The scanning electron microscope (SEM) figure is shown in Fig. 9(a). Meanwhile, since the panda-type structure is commonly used to suppress the polarization mode coupling arising in the polarization maintaining single mode fiber (SMF), we also design PM-FMF with panda-type structure. Panda-FMF is fabricated by a multi-step process, involving drilling holes into a solid preform and inserting pre-fabricated borosilicated stress rod. With almost the same fiber drawing technique, the panda-FMF with core cladding RI difference of 5.0 × 10^−3^ can be obtained, while the corresponding SEM figure is shown in Fig. 9(b). The core and cladding diameters of panda-FMF are 21 μm and 125 μm, respectively. The distance between the centre of stress-applied parts and fiber core is 30 μm. We are able to take advantage of finite element mode solver (COMSOL5.2) to obtain the field distribution and its corresponding mode effective index. For the e-FMF, there are total 10 guided spatial and polarization modes, including LP_01x_, LP_01y_, LP_11ax_, LP_11ay_, LP_11bx_, LP_11by_, LP_21ax_, LP_21ay_, LP_21bx_, and LP_21by_. Similarly, for the panda-FMF, the 11 guided spatial and polarization modes are LP_01x_, LP_01y_, LP_11ax_, LP_11bx_, LP_11by_, LP_11ay_, LP_21x_, LP_21bx_, LP_02x_, LP_31ax_, and LP_31bx_, respectively. For the ease of understanding, the spatial distribution of LP_11_ and LP_21_ modes with the corresponding mode spacing to the LP_01x_ mode are shown in the Fig. 10. When the fiber structure varies from conventional circular core, mode degeneration is broken and LP modes become true modes in PM-FMFs, in contrast with c-FMF^25^. When twisting of these PM-FMF happens, two kinds of coupling can be determined. Specifically, spatial mode coupling means the coupling between LP_11a_ and LP_11b_, while polarization mode coupling occurs between LP_11x_ and LP_11y_. Consequently, for the e-FMF, the off-diagonal coefficients in Eq. (1) are of about 0.09 for spatial mode coupling and 0.71 for polarization mode coupling, respectively. Obviously, the spatial mode coupling is trivial enough, while the polarization mode coupling is non-negligible. When the e-FMF is twisted, spatial distribution of the launched LP_11_ mode can be maintained, but the linear polarization may vary from time to time. Similarly, spatial and polarization mode coupling of panda-FMF are calculated as 6.9 × 10^−4^ and 9.2 × 10^−8^. Both couplings are extremely weak, indicating that the spatial pattern can be well preserved with linear polarization, when the panda-FMF is twisted.
